# Redd1 knockdown prevents doxorubicin-induced cardiac senescence

**DOI:** 10.18632/aging.202972

**Published:** 2021-05-06

**Authors:** Pianpian Huang, Lijuan Bai, Lihua Liu, Jun Fu, Kefei Wu, Hongxia Liu, Yun Liu, Benming Qi, Benling Qi

**Affiliations:** 1 Department of Geriatrics, Union Hospital, Tongji Medical College, Huazhong University of Science and Technology, Wuhan, Hubei 430022, China; 2 Department of Geriatrics, Wuhan No.1 Hospital, Wuhan, Hubei 430022, China; 3 Department of Radiology, Wuhan No.1 Hospital, Wuhan, Hubei 430022, China; 4 Department of Otorhinolaryngology, First People’s Hospital of Yunnan Province, Kunming, Yunnan 650000, China

**Keywords:** doxorubicin, cardiomyocyte senescence, redd1, p38 MAPK, NF-κB

## Abstract

Regulated in development and DNA damage response-1 (Redd1) is a stress-response gene that is transcriptionally induced by diverse stressful stimuli to influence cellular growth and survival. Although evidence suggests that aging may drive Redd1 expression in skeletal muscles, the expression patterns and functions of Redd1 in senescent cardiomyocytes remain unspecified. To address this issue, *in vitro* and *in vivo* models of cardiomyocyte senescence were established by administration of doxorubicin (Dox). Redd1 overexpression and knockdown was achieved in cultured H9c2 cardiomyocytes and mouse tissues using, respectively, lentivirals and adeno-associated virus 9 (AAV9) vectors. In the hearts of both aged (24 months old) and Dox-treated mice, as well as in Dox-exposed H9c2 cardiomyocytes, high Redd1 expression accompanied the increase in both cellular senescence markers (p16^INK4a^ and p21) and pro-inflammatory cytokine expression indicative of a stress-associated secretory phenotype (SASP). Notably, Redd1 overexpression accentuated, whereas Redd1 silencing markedly attenuated, Dox-induced cardiomyocyte senescence features both *in vitro* and *in vivo*. Notably, AAV9-shRNA-mediated Redd1 silencing significantly alleviated Dox-induced cardiac dysfunction. Moreover, through pharmacological inhibition, immunofluorescence, and western blotting, signaling pathway analyses indicated that Redd1 promotes cardiomyocyte senescence as a downstream effector of p38 MAPK to promote NF-kB signaling via p65 phosphorylation and nuclear translocation.

## INTRODUCTION

Cardiovascular diseases (CVDs) account for over 30% of deaths worldwide and are increasingly prevalent in the aging population [1, 2]. Indeed, aging is regarded as a major independent risk factor for common heart diseases including heart failure [3]. Significant pathophysiological changes occur in the aging heart. These include interstitial fibrosis and cardiomyocyte hypertrophy, leading to increased ventricular stiffness and a reduction in diastolic, rather than systolic, function [4]. Therefore, a deeper understanding of the molecular mechanisms of cardiac aging will help design novel interventions to prolong health span and decrease CVD-related morbidity and mortality in the elderly [5–9].

Accumulating evidence indicates that senescence, a process defined by a range of stress-related responses that occur at both the cellular and organismal level, links a variety of age-related pathologies [10–12]. Cellular senescence is classically defined as the irreversible loss of division potential of mitotic cells, usually accompanied by a senescence-associated secretory phenotype (SASP) [13]. In turn, organismal aging is characterized by both impaired ability to respond to stressors and homeostatic imbalance, which compromise normal tissue and organ functioning [14]. Since cellular senescence largely forms the basis of organismal aging, cell-based models are useful for studying the mechanisms underlying organismal aging [15]. A defining feature of cellular senescence is the arrest of cell cycle progression, caused by telomere shortening or stress-induced premature senescence, leading to a flattened, enlarged cell morphology and lysosomal activation, verified experimentally by assessment of senescence-associated β-galactosidase (SA-β-gal) staining [16–18]. Post-mitotic cardiomyocyte senescence has been reported to contribute to myocardial remodeling, the basic pathological alteration in myocardial diastolic dysfunction [19]. Therefore, therapeutic modulation of cardiomyocyte senescence represents a promising strategy to prevent or delay aging-related cardiac remodeling and dysfunction.

Regulated in development and DNA damage response-1 (Redd1), also known as DNA Damage-Inducible Transcript 4 (DDIT4), is a stress-response gene that is transcriptionally induced in various types of cells by stressful stimuli such as DNA damage, hypoxia, and energy deficits [20, 21]. Redd1 is a potent inhibitor of mammalian Target of Rapamycin (mTOR) Complex-1 (mTORC1), and its expression influences a wide spectrum of cellular processes and biological functions, such as cell cycle, autophagy, energy homeostasis, and inflammation [22–25]. A regulatory role for Redd1 was reported in phenylephrine-induced cardiac hypertrophy [26] and myocardial ischemia/reperfusion injury [27, 28]. However, whether and how Redd1 regulates cardiomyocyte senescence during aging remains to be studied. Therefore, this study compared cardiac Redd1 expression in young and old mice and explored its role on cardiomyocyte senescence using *in vitro* and *in vivo* models of doxorubicin (Dox)-induced cardiotoxicity.

## RESULTS

### Cardiac Redd1 expression increases with advancing age

**Figure 1:**
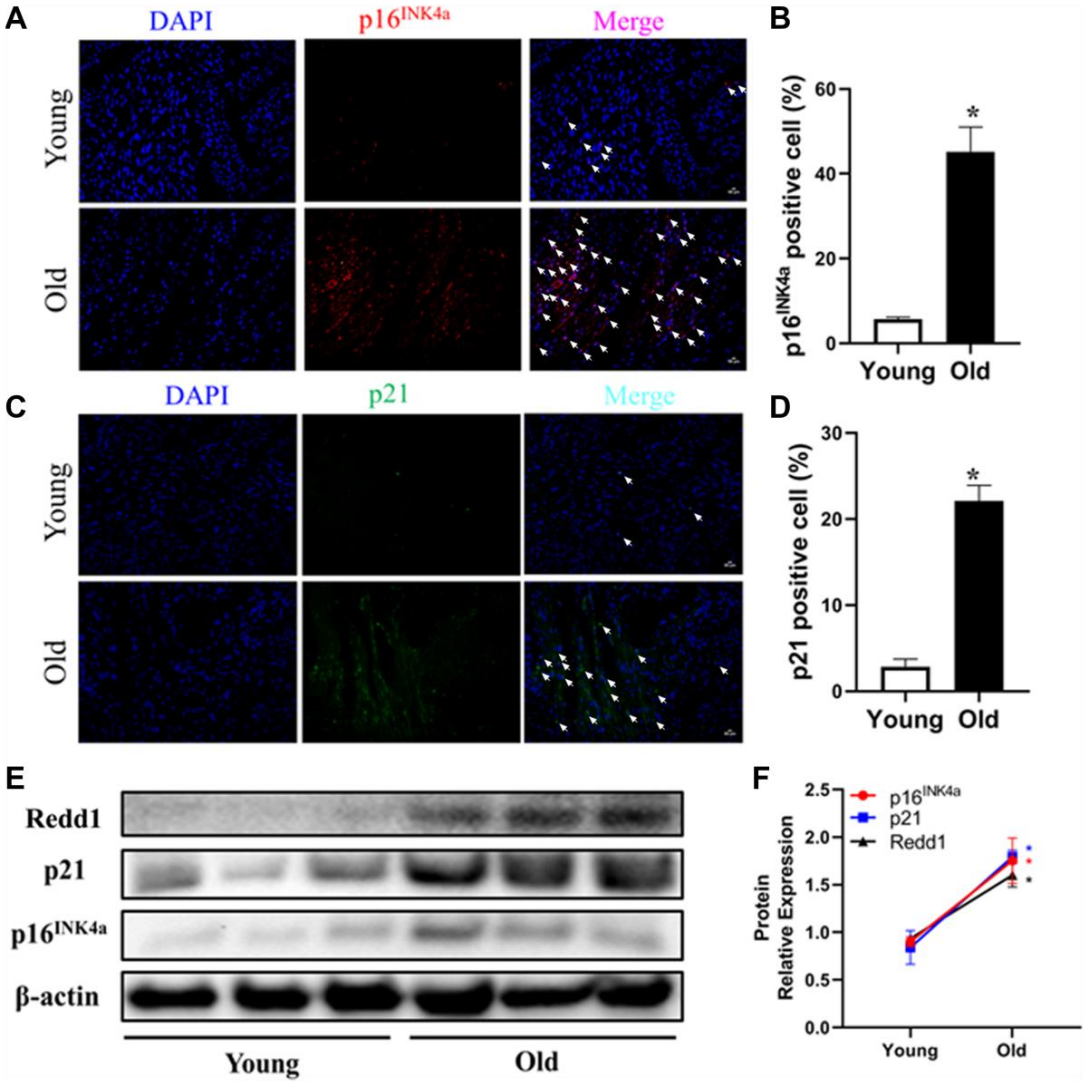
**Cardiac Redd1 expression increases with advancing age.** (**A**, **B**) Representative photomicrographs and quantitative analysis of myocardial p16^INK4a^ immunofluorescent staining in young group mice (3~4 months old) and old group mice (24 months old) (*n* = 6 per group). (**C**, **D**) Representative photomicrographs of myocardial p21 immunofluorescent staining and quantitative expression analysis in young group mice and old group mice (*n* = 6 per group). (**E**, **F**) Western blotting detection and quantification of Redd1, p16^INK4a^, and p21 protein expression in the heart of young group mice and old group mice (*n* = 6–8 per group). Data are mean ± SEM. ^*^p < 0.05 vs. young group.

Cellular senescence, considered a hallmark of organismal aging, is characterized by elevated expression of senescence marker genes [29]. To investigate the effect of aging on cardiac cell senescence, the expression of two senescence-related cell-cycle regulators (i.e., p16^INK4a^ and p21) was evaluated in heart tissues from young (3–4 months old) and old (24 months old) male C57BL/6 mice. While p16^INK4a^ and p21 expression was barely detectable in heart tissues from young mice, extensive immunoreactivity was observed for both markers in aged heart tissues (Figure 1A–1D). Accompanying these changes, the expression of Redd1 was also clearly increased in the hearts of older, but not young, animals (Figure 1E and 1F). These data suggest that Redd1 may be involved in the process of cardiac aging.

### Dox exposure upregulates Redd1 expression in both cardiac tissue and cultured cardiomyocytes

**Figure 2:**
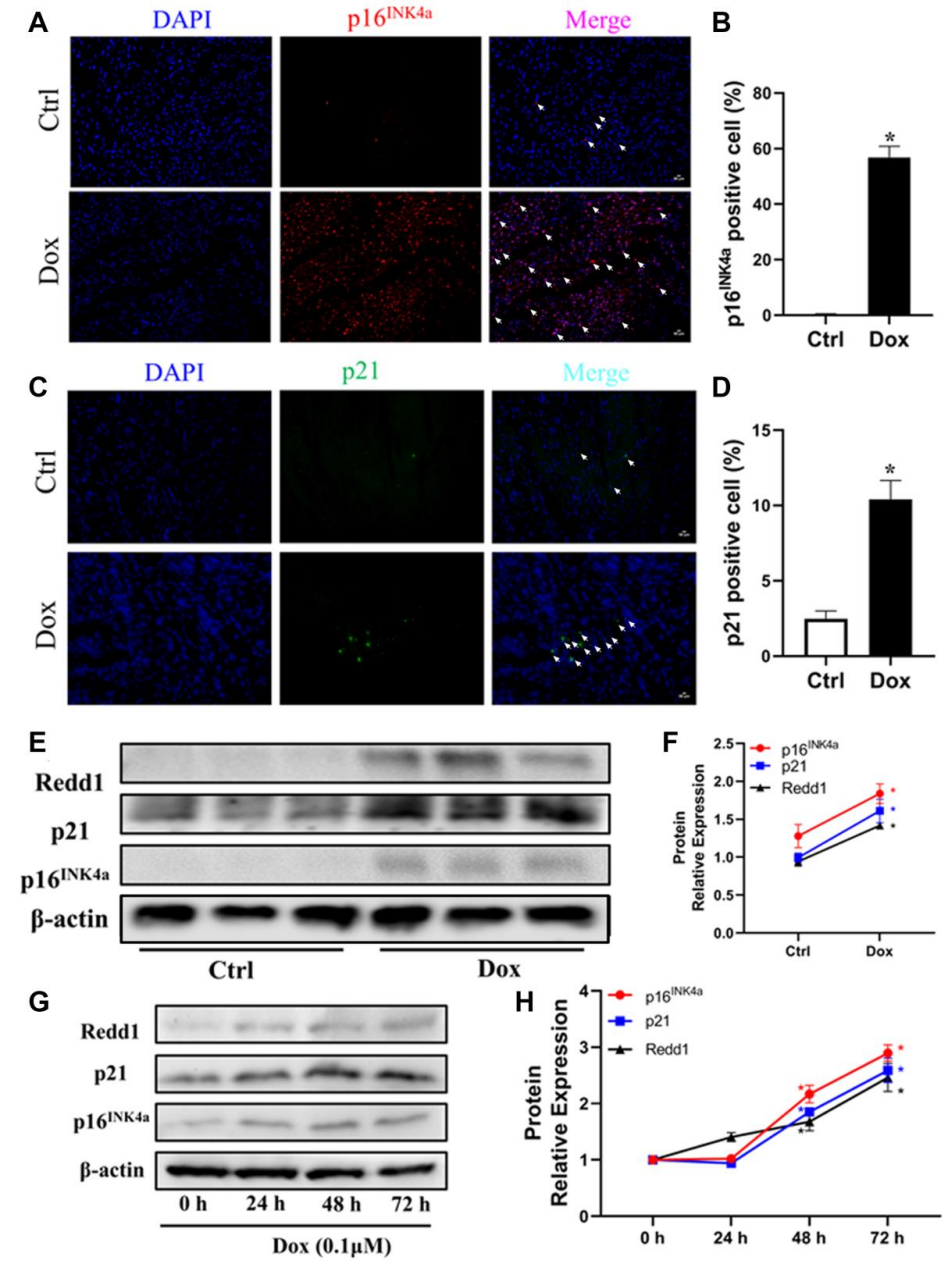
**Dox exposure upregulates Redd1 expression in both cardiac tissue and cultured cardiomyocytes.** (**A**, **B**) Representative photomicrographs and quantitative analysis of cardiac p16^INK4a^ immunofluorescent staining in control and Dox-treated mice (*n* = 6 per group). (**C**, **D**) Representative photomicrographs and quantitative analysis of cardiac p21 immunofluorescent staining in control and Dox-treated mice (*n* = 6 per group). (**E**, **F**) Western blotting detection and quantification of Redd1, p16^INK4a^, and p21 expression in cardiac tissue from control and Dox-treated mice (*n* = 6–8 per group). (**G**, **H**) Time-course expression and quantification analysis of Redd1, p16^INK4a^, and p21 protein levels in cultured H9c2 cardiomyocytes treated with Dox (0.1 μM) (*n* = 3 samples per group). Data are mean ± SEM. ^*^p < 0.05 vs. control group.

To further elucidate the relationship between Redd1 expression and cardiac senescence, we examined potential changes in cardiac Redd1 expression in a mouse model of Dox-induced cardiomyopathy. Immunolabeling and western blot assays showed that the expression of p16^INK4a^, p21, and Redd1 was significantly increased in myocardial tissues from young adult mice exposed to a 2-week Dox regimen (Figure 2A–2F). To verify these data, we next administered Dox to rat H9c2 cardiomyocytes *in vivo* to induce a senescence phenotype. Consistent with the results obtained *in vivo*, at a relatively low dose of 0.1 μM, increased expression of p16^INK4a^, p21, and Redd1 suggested effective induction of a senescent phenotype in Dox-treated cardiomyocytes (Figure 2G and 2H). These data indicated that Redd1 overexpression parallels characteristic senescence-related molecular changes induced by either physiological aging or Dox.

### Redd1 suppression attenuates Dox-induced senescence in cardiomyocytes

**Figure 3:**
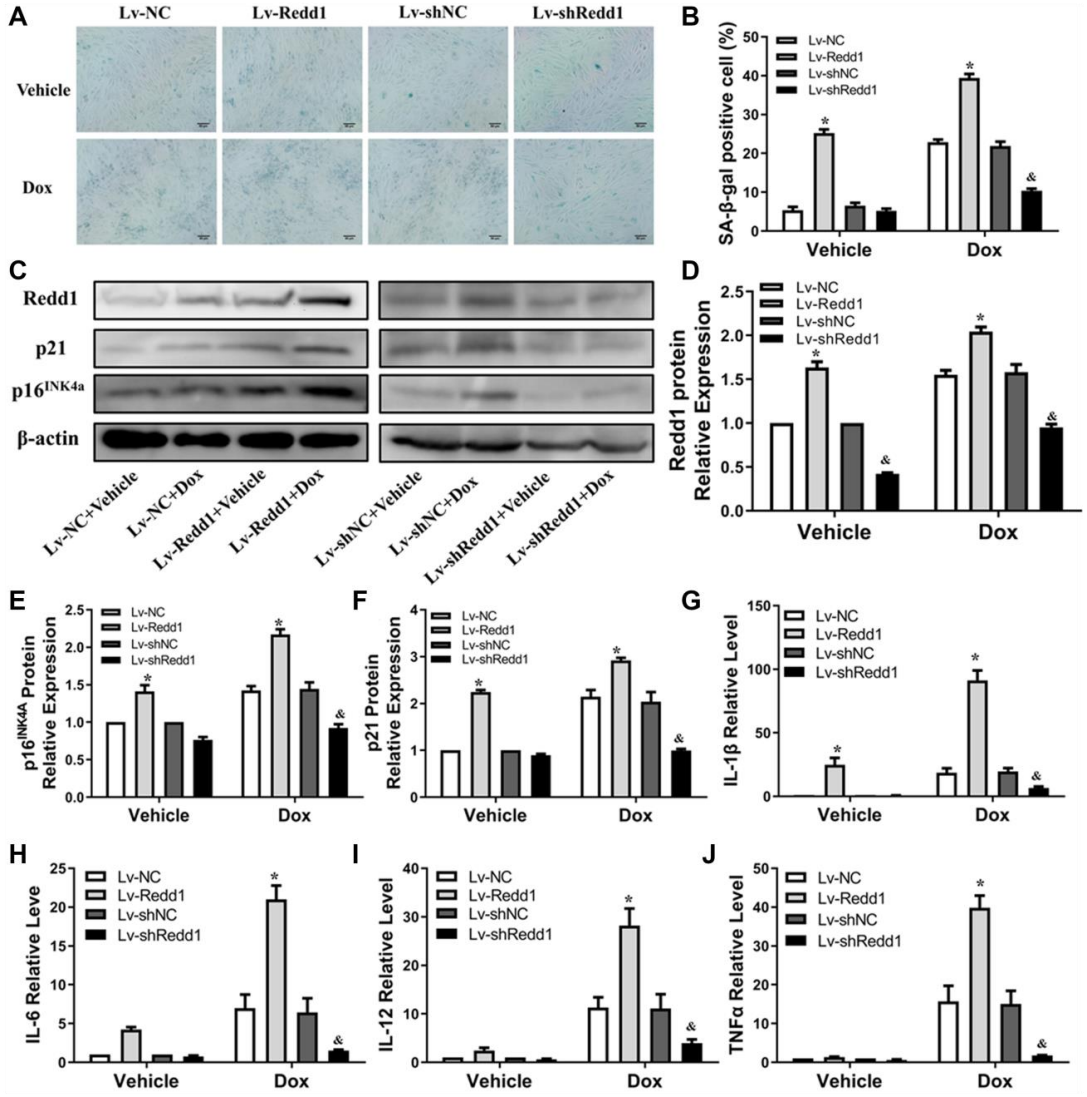
**Redd1 suppression attenuates Dox-induced senescence in cardiomyocytes.** (**A**) Representative images of SA-β-gal staining in Dox-challenged H9c2 cardiomyocytes following Redd1 knockdown or overexpression. (**B**) Quantitative analysis of SA-β-gal activity; the number of SA-β-gal positive cells observed in at least 6 separate microscopic fields is indicated (*n* = 6 per group). ^*^p < 0.05 vs. the Lv-NC group. ^&^*p* < 0.05 vs. the Lv-shNC group. (**C**–**F**) Effects of Redd1 overexpression and knockdown on p16^INK4a^ and p21 expression in Dox-challenged H9c2 cardiomyocytes (*n* = 3 per group). ^*^p < 0.05 vs. the Lv-NC group. ^&^*p* < 0.05 vs. the Lv-shNC group. (**G**–**J**) Effect of Redd1 overexpression and knockdown on IL-1β, IL-6, IL-12, and TNFα mRNA in Dox-stimulated H9c2 cardiomyocytes (*n* = 3 per group). Data are mean ± SEM. ^*^p < 0.05 vs. the Lv-NC group. ^&^*p* < 0.05 vs. the Lv-shNC group.

To define the role of Redd1 in Dox-induced cardiomyocyte senescence *in vitro*, we applied a lentivirus (Lv)-based approach to modify the expression of Redd1 in cultured rat H9c2 cardiomyocytes. Predictably, SA-β-gal staining was significantly elicited in Dox-treated cardiomyocytes and was further enhanced by Lv-Redd1-mediated upregulation of Redd1 (Figure 3A and 3B). By contrast, downregulation of Redd1 expression via transduction with Lv-shRedd1 partly abrogated the increase in SA-β-gal staining. Paralleling these changes, Redd1 overexpression and silencing increased and decreased, respectively, Dox-induced expression of p16^INK4a^ and p21 (Figure 3C–3F). To further verify the role of Redd1 in cardiomyocyte senescence, we assessed the SASP of Dox-treated H9c2 cells by quantifying mRNA levels of IL-1β, IL-6, IL-12, and TNFα. As shown in Figure 3G–3J, the expression of these pro-inflammatory cytokines was remarkably increased by Dox treatment. In line with the findings described so far, the SASP was exacerbated and attenuated, respectively, by upregulation and repression of Redd1. These results revealed that Redd1 plays a vital role in promoting Dox-induced premature senescence in cardiomyocytes.

### Dox exposure upregulates Redd1 in cardiomyocytes via p38 MAPK signaling

**Figure 4:**
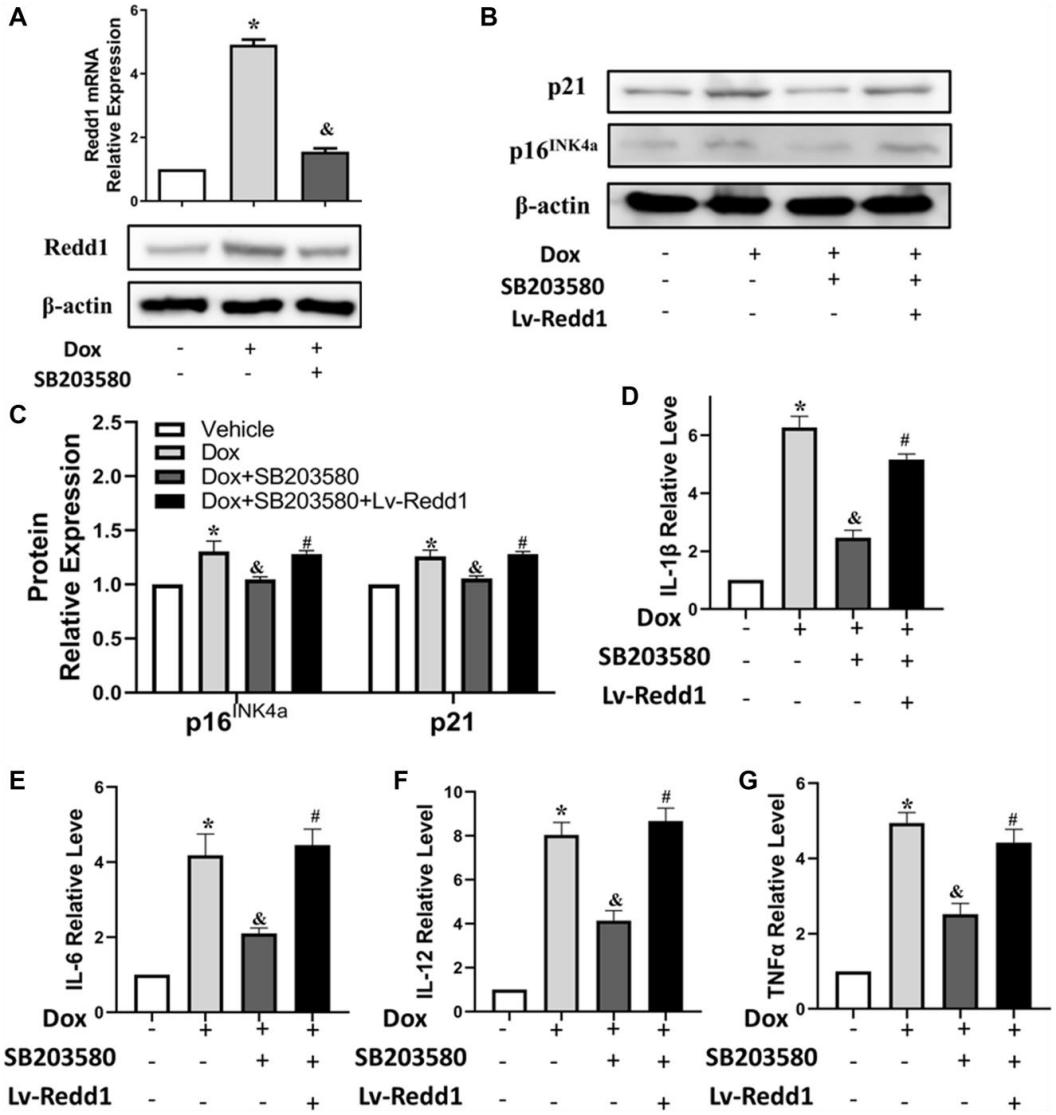
**Dox exposure upregulates Redd1 in cardiomyocytes via p38 MAPK signaling.** (**A**) Detection of Redd1 expression by q-PCR and western blotting in Dox-treated H9c2 cardiomyocytes pretreated with the p38 MAPK inhibitor SB203580 (2 μM). (*n* = 4 per group). (**B**, **C**) Effect of Redd1 overexpression on p16^INK4a^ and p21 protein levels in Dox-challenged H9c2 cardiomyocytes pretreated with SB203580 (*n* = 4 per group). (**D**–**G**) Effect of Redd1 overexpression on IL-1β, IL-6, IL-12, and TNFα mRNA in Dox-challenged H9c2 cardiomyocytes pretreated with SB203580 (*n* = 4 per group). Data are mean ± SEM. ^*^p < 0.05 vs. control group. ^&^*p* < 0.05 vs. Dox group. ^#^*p* < 0.05 vs. Dox+SB203580 group.

Dox acts through multiple pathways leading to cardiomyocyte senescence. Based on previous findings linking p38 MAPK activity with both Dox-induced cardiomyocyte senescence [30] and Redd1 expression [31], we tested the effect of SB203580, a highly selective p38 MAPK inhibitor, on Redd1 expression in Dox-treated H9c2 cardiomyocytes. As shown in Figure 4A, pretreatment with SB203580 (2 μM) completely abrogated Dox-induced Redd1 upregulation. Furthermore, the expression of both p16^INK4a^ and p21 was also blunted by pre-exposure to the p38 MAPK inhibitor, and partly rescued by concomitant transduction with Lv-Redd1 to achieve Redd1 overexpression (Figure 4B and 4C). Subsequently, qPCR analysis was performed to assess the effect of p38 MAPK inhibition on the cardiomyocytes’ SASP. As illustrated in Figure 4D–4G, SB203580 almost fully reversed the significant increase in SASP-related cytokines induced by Dox. However, the effect of SB203580 was abolished after transduction of H9c2 cells with Lv-Redd1. These results suggest that p38 MAPK acts as an upstream activator of Redd1 during Dox-induced senescence in cardiomyocytes.

### Redd1 expression activates NF-κB signaling in senescent cardiomyocytes

**Figure 5:**
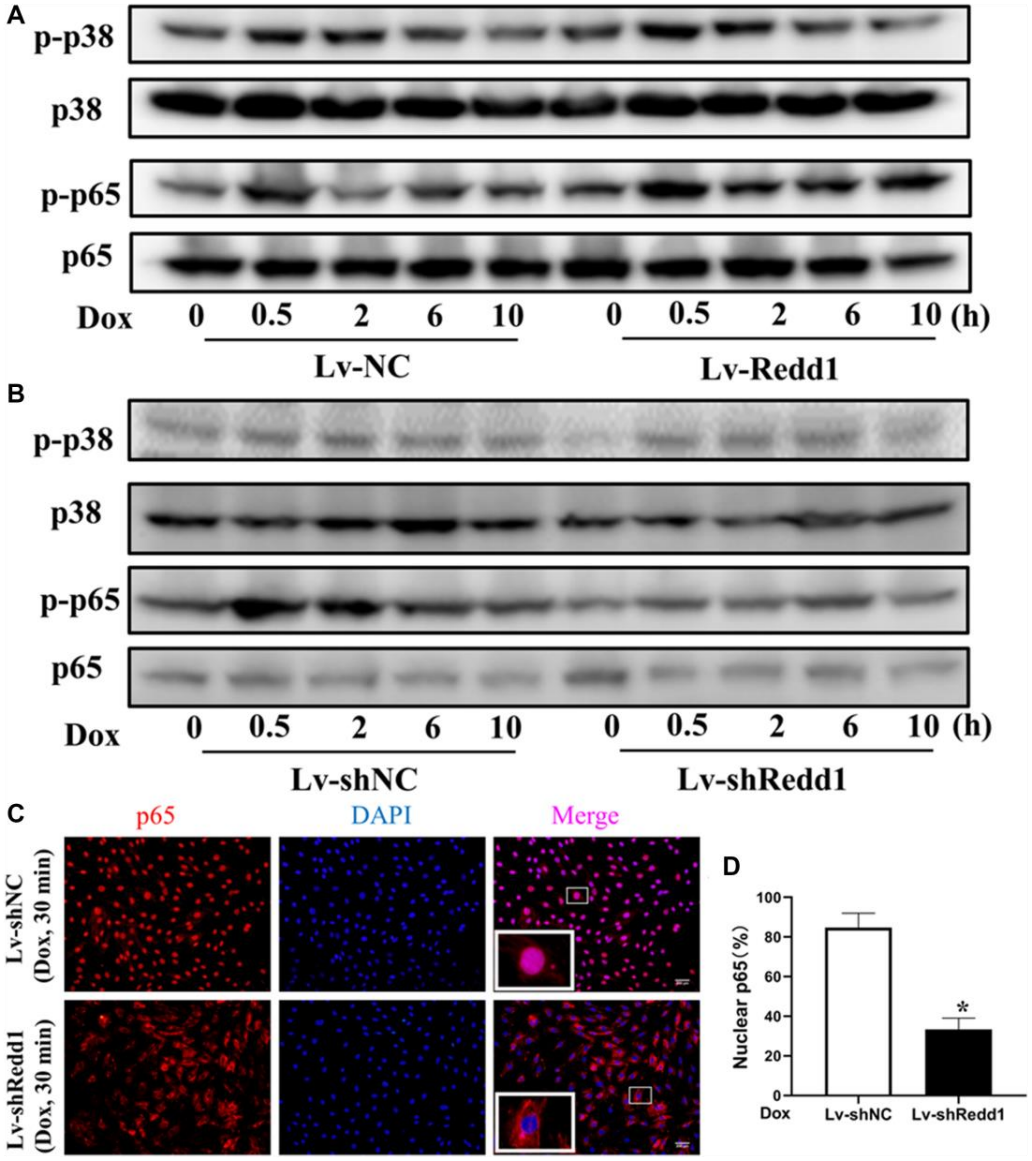
**Redd1 expression activates NF-κB signaling in senescent cardiomyocytes.** (**A**, **B**) Western blot analysis of total and phosphorylated p65 and p38 MAPK in Dox-treated H9c2 cardiomyocytes following Redd1 overexpression and knockdown. (**C**) Immunofluorescent detection of nuclear translocation of p65 in Dox-treated H9c2 cardiomyocytes following Redd1 overexpression and knockdown. (**D**) Quantification of data from p65 nuclear translocation assays (*n* = 4 per group). ^*^p < 0.05 vs. Lv-shNC group. Data are mean ± SEM. ^*^p < 0.05 vs. control group. ^&^*p* < 0.05 vs. Lv-Redd1 group.

Considering the major contributions of the NF-κB and p38 MAPK pathways to the mechanisms determining cellular senescence responses, we next examined the impact of Redd1 expression on the phosphorylation of critical signaling proteins within these pathways. As shown in Figure 5A and 5B, the phosphorylation status of p65 and p38 was enhanced in H9c2 cells subjected to 30-min Dox incubation. After Redd1 silencing or overexpression, the phosphorylation of p65, but not p38, was affected. Specifically, Redd1 overexpression markedly increased p65 phosphorylation (Figure 5A and 5B), whereas immunofluorescence assays indicated that Redd1 silencing inhibited Dox-induced p65 nuclear translocation (Figure 5C and 5D). Collectively, these data suggest that Redd1 acts as a positive regulator of the NF-κB signaling pathway to promote a senescence-associated phenotype in cardiomyocytes.

### Redd1 expression enhances Dox-induced cardiac senescence *in vivo*

**Figure 6:**
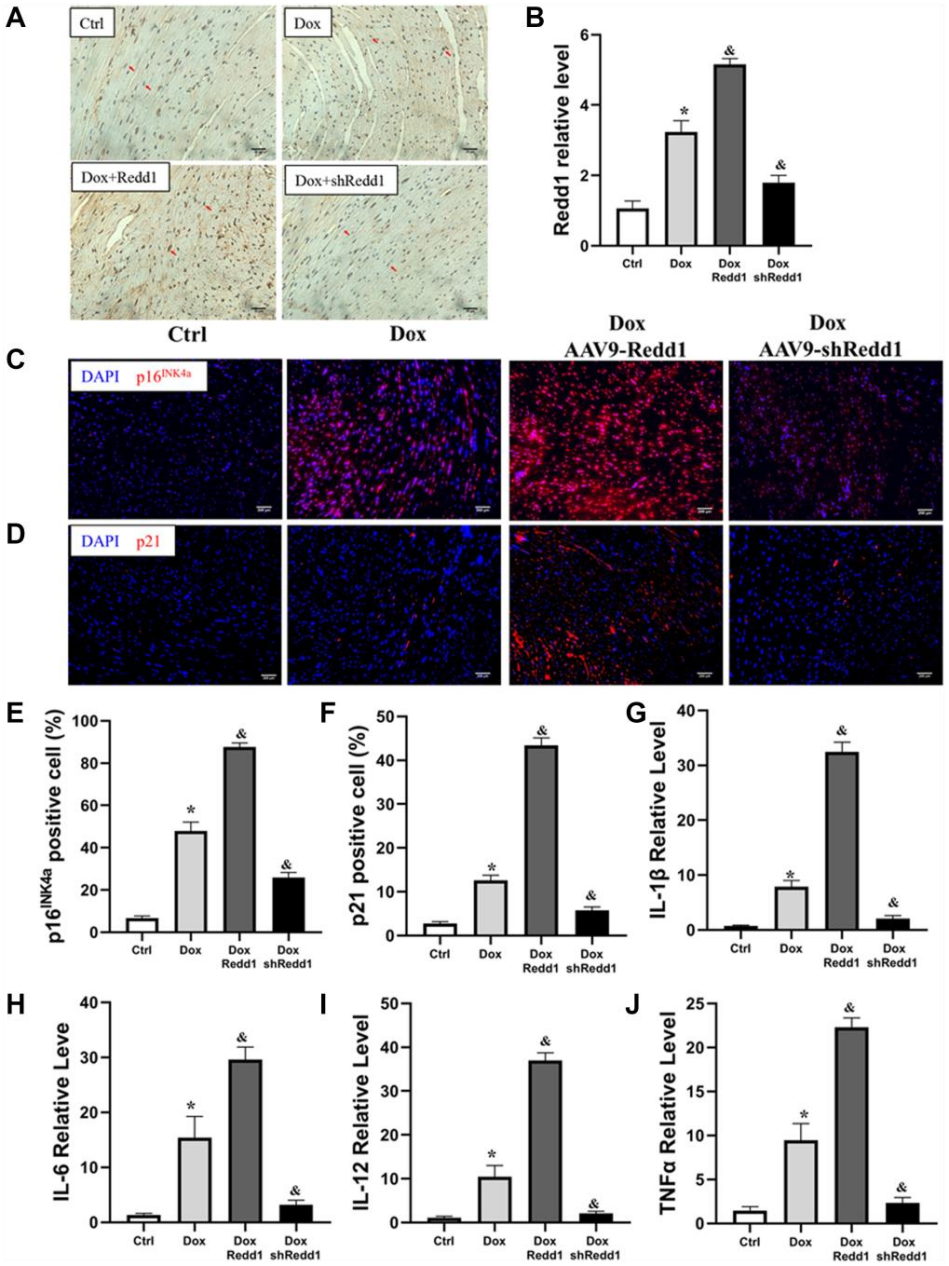
**Redd1 expression enhances Dox-induced cardiac senescence *in vivo*.** (**A**, **B**) Representative images and corresponding quantitative expression analysis of Redd1 immunostaining in mouse heart tissues (*n* = 6 mice per group). (**C**, **D**) Immunofluorescence analysis of the effect of Redd1 overexpression and knockdown on p16^INK4a^ and p21 expression in cardiac samples from Dox-treated mice. (**E**, **F**) Quantification of p16^INK4a^ and p21 expression data (*n* = 6–8 per group). (**G**–**J**) Effect of Redd1 overexpression and knockdown on IL-1β, IL-6, IL-12, and TNFα mRNA in cardiac tissue from Dox-treated mice (*n* = 6–8 per group). Data are mean ± SEM. ^*^p < 0.05 vs. the AAV9-NC group. ^&^*p* < 0.05 vs. the AAV9-shNC group.

To verify the pro-senescence effect of Redd1 on cardiac cells *in vivo*, Redd1 was overexpressed or repressed in mouse tissues by delivering, via tail vein injection and 4 weeks before Dox administration, AAV9 viral vectors encoding, respectively, the Redd1 gene or a Redd1-targeting shRNA. The efficiency of these approaches was confirmed by immunohistochemical analysis of cardiac tissues, which showed also that Dox treatment increased cardiac Redd1 levels relative to saline-injected controls (Figure 6A and 6B). In full agreement with our *in vitro* findings, Redd1 overexpression further exacerbated the increase in cardiac p16^INK4a^ and p21 expression induced by Dox, while the opposite effect was observed after Redd1 silencing (Figure 6C–6F). Moreover, qPCR assays showed that Dox treatment increased the SASP in cardiac tissues, and this phenomenon was remarkably enhanced and repressed by Redd1 overexpression and knockdown, respectively (Figure 6G–6J). Collectively, these data indicated that Redd1 affected the induction of senescence-associated phenotypes in the hearts of Dox-treated mice.

### Modulation of Redd1 expression impacts Dox-induced cardiac alterations *in vivo*

**Figure 7:**
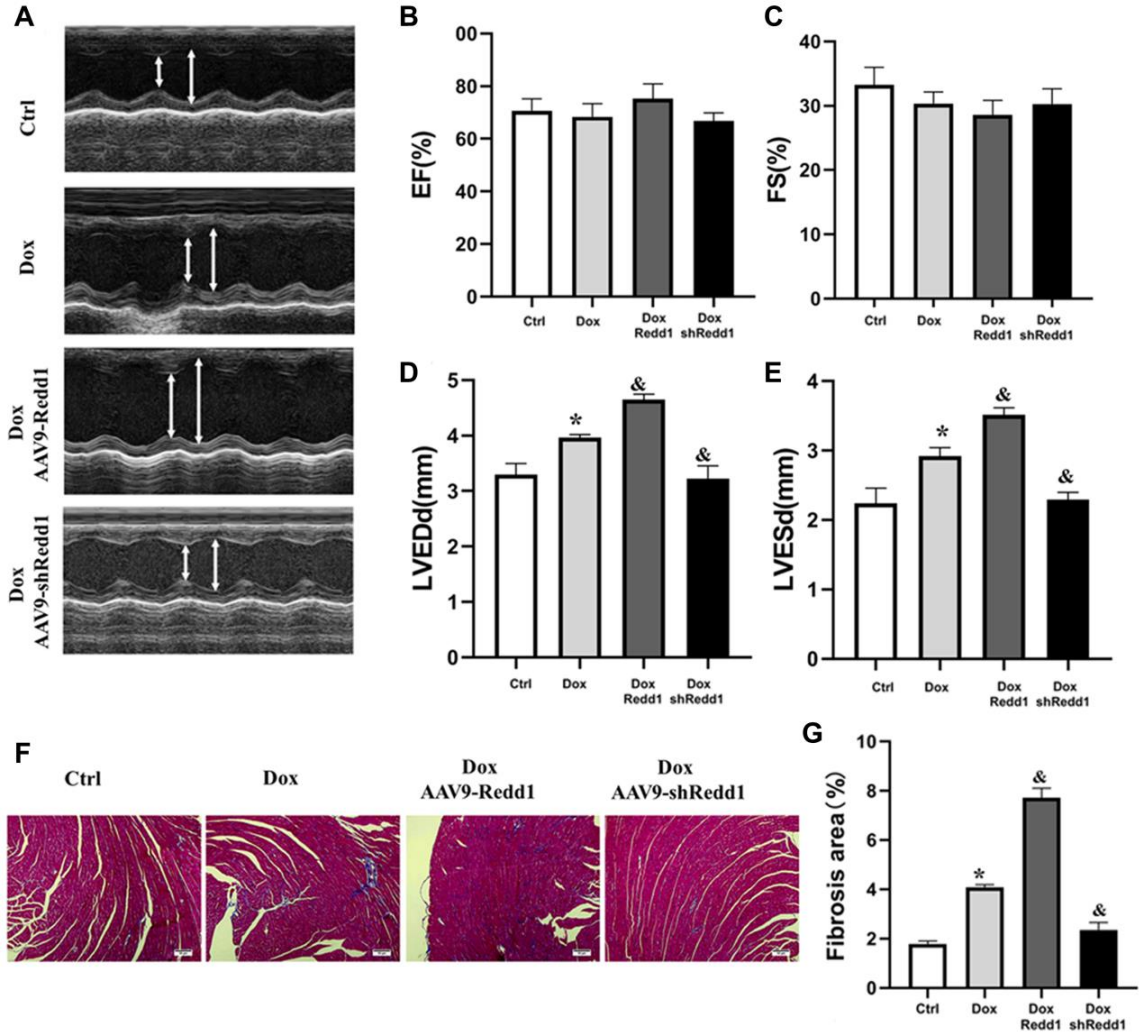
**Modulation of Redd1 expression impacts Dox-induced cardiac alterations *in vivo*.** (**A**) Representative echocardiography photographs depicting left ventricular function in the different groups of mice. (**B**–**E**) Analysis of the effects of Redd1 overexpression and knockdown on parameters of cardiac function in Dox-treated mice (*n* = 6–8 per group). (**F**) Effect of Redd1 overexpression and knockdown on cardiac fibrosis induced by Dox administration *in vivo*, as determined by Masson’s trichrome staining. (**G**) Quantitative analysis of Masson’s trichrome staining results (*n* = 6–8 per group). Data are mean ± SEM. ^*^p < 0.05 vs. the AAV9-NC group. ^&^*p* < 0.05 vs. the AAV9-shNC group.

Next, we sought to determine whether altering Redd1 expression could modulate cardiac function in Dox-treated mice. To this end, echocardiography images were obtained to observe changes in left ventricular systolic and diastolic function (Figure 7A). In Dox-treated mice, left ventricular end diastolic diameter (LVEDd) and left ventricular end-systolic diameter (LVESd) were significantly increased, whereas left ventricular ejection fraction (LVEF) and left ventricular fractional shortening (LVFS) showed no obvious change (Figure 7B and 7C). In turn, both LVEDd and LVESd were further augmented by AAV9-Redd1 and decreased instead by AAV9-shRedd1 administration (Figure 7D and 7E). In parallel experiments, alterations in cardiac function were also confirmed in aged mice (Supplementary Figure 1). To evaluate whether cardiac fibrosis was also impacted by Redd1 expression, we examined collagen expression in heart samples using Masson’s trichrome staining. The results showed that Dox treatment increased collagen deposition in the heart, and this effect was enhanced and attenuated, respectively, by Redd1 overexpression and knockdown (Figure 7F and 7G). These results demonstrated that increased Redd1 expression contributes to Dox-mediated cardiac dysfunction and suggest that strategies aimed at decreasing endogenous cardiac Redd1 levels may help preserve normal functioning in the aged heart.

## DISCUSSION

Cardiac remodeling and dysfunction are normal consequences of aging, manifested at the molecular level by activation of senescence-associated pathways in cardiomyocytes. In this study, we found that Redd1 was highly expressed in the hearts of aged mice and its levels were intimately correlated with those of common markers of cellular senescence. To explore the role of Redd1 in cardiomyocyte senescence, the Dox-induced cardiomyocyte senescence model was applied to *in vitro* and *in vivo* experimental setups. Our results showed that the Dox administration protocols reproduced, both adequately and within a relatively short period of time, main senescence features in both cultured and *in situ* rodent cardiomyocytes.

The current study directly identified Redd1 as a key determinant of cardiomyocyte senescence. Moreover, in our mouse model of Dox-induced cardiomyopathy, we found that AAV9-mediated Redd1 overexpression aggravated Dox-induced cardiac function, SASP-related cytokine production, and the expression of senescence markers (i.e., p16^INK4a^ and p21) in cardiomyocytes. Accordingly, silencing endogenous Redd1 with a specific shRNA markedly reduced myocardial senescence indicators (i.e., SASP, p16^INK4a^, and p21 expression) and exerted a cardio-protective effect *in vivo*.

Redd1 expression is stimulated by cellular stress and influences a wide spectrum of cellular processes mainly through inhibition of mTOR signaling [22–25]. It was reported that Redd1 expression in the hippocampus increases markedly during normal aging, suggesting that Redd1 is an aging-associated factor [32]. Current evidence suggests that Redd1 may play an important role during cellular senescence. For instance, it was reported that metformin induces cell cycle arrest by increasing Redd1 expression in a p53-dependent manner [33], whereas senescence in prostate cancer cells can be promoted by AMPK-mediated Redd1 expression [34]. Although several studies demonstrated that Redd1 is upregulated during skeletal muscle aging [35, 36], there are no available studies, to our knowledge, on the role of Redd1 in myocardial aging. Our results indicated that Redd1 expression was increased both in the aging mouse heart and in the myocardium of young adult mice following Dox exposure. We noted that the expression of Redd1 increased in a time-dependent manner in Dox-stimulated, senescent cardiomyocytes, suggesting that Redd1 might be involved in the process of senescence-induced cardiac damage.

A regulatory role for Redd1 in insulin signaling may partly explain its pro-senescence effects in cardiomyocytes. Using Redd1 knockout mice, Williamson and collaborators reported that Redd1 silencing decreased insulin-stimulated growth signaling responses in skeletal muscle [37–39]. Meanwhile, research has shown that reduced insulin signal transduction improved heart performance in *Drosophila melanogaster* and reduced, in mice, cardiomyocyte dysfunction related to aging [40, 41]. We showed that Redd1 was upregulated in the hearts of both aged and Dox-treated mice, as well as in Dox-treated H9c2 rat cardiomyocytes. However, none of the current, commonly used *in vivo* or *in vitro* cardiac aging models can fully recapitulate all the phenotypes of natural aging. Previous research provided evidence of the association of Redd1 with age-related diseases [32–34, 42–45]. Several data collected in this work converged on supporting the contribution of Redd1 to cardiomyocyte senescence and senescence-related alterations in heart structure and function. Regulation of aging markers following Redd1 knockdown in mice highly suggests that chronic Redd1 inhibition could provide a protective effect during natural cardiac aging. Thus, studies on Redd1 knockdown mice are necessary to fully elucidate the role of Redd1 in the natural cardiac aging process.

Cellular senescence is classically defined as an irreversible loss of division potential of mitotic cells [13]. The majority of cardiomyocytes are post-mitotic, withdrawing from the cell cycle shortly after birth. It has been controversial whether there is a senescent phenotype in cardiomyocytes. However, numerous studies have reported the presence of senescent cardiomyocytes, characterized by telomere shortening and positive SA-β-gal staining [46, 47]. Similarly, our *in vitro* experiments confirmed, via SA-β-gal staining, the senescence phenotype of cardiomyocytes. Recently, Anderson et al. demonstrated that the cardiomyocyte senescence-like phenotype is a feature of normal physiological aging in both mice and humans, and provided a novel mechanistic model by which senescence occurs in rarely dividing/post-mitotic tissues [19]. Our *in vivo* experiments also confirmed main senescence features in cardiomyocytes, namely high p16^INK4a^ and p21 expression, as well as a SASP defined by increased production of pro-inflammatory mediators. Paradoxically, a previous study reported no changes in p16^INK4a^ and p21 mRNA levels in cardiac and skeletal muscles in 27-month-old mice [48]. However, Krishnamurthy et al. reported increased mRNA expression for both p16^Ink4a^ and p21 in the heart of 26-month-old mice, compared with younger animals [49]. Since mRNA quantification can be affected by a variety of conditions, including the quality of the RNA extraction process and of the PCR primers used, protein expression data may arguably be more persuasive. Indeed, and consistent with our results, other previous studies demonstrated that p16^Ink4a^ and p21 protein levels were upregulated in aged heart tissue [50, 51].

Previous results indicated that cellular senescence may slow down tumor cell proliferation, migration, invasion, and metastasis [52, 53]. Thus, therapeutic approaches that induce tumor cell senescence may be useful to inhibit tumor progression and to extend the lifespan of cancer patients [54]. However, in healthy cells, senescence is associated with organ/tissue deterioration and impaired functioning, which shortens lifespan by increasing the likelihood of pathological outcomes [54, 55]. Pro-inflammatory, senescent cardiomyocytes are activated to induce the proliferation of fibroblasts. This leads to interstitial fibrosis and hypertrophy of cardiomyocytes [19], i.e., the defining characteristic of pathological cardiac remodeling and dysfunction [56]. Our study indicated that Redd1 expression was associated with both fibrosis and acquisition of a pro-inflammatory SASP by cardiomyocytes. Therefore, it is likely that the role of Redd1 in promoting senescence-induced cardiac alterations is mediated, at least in part, by its ability to induce excessive inflammation and fibrosis.

We found that Dox treatment induced Redd1 upregulation and senescence responses via p38 MAPK activation in cultured cardiomyocytes. Activation of p38 MAPK contributes to the induction of senescence-associated cell cycle inhibitor p16^INK4a^ and plays an important role in cellular senescence mechanisms triggered by different stimuli [57–59]. There are four p38 MAPK isoforms (p38α, p38β, p38γ, and p38δ) activated by a variety of cellular stressors. p38 MAPK was reported to be activated in Dox-stimulated senescent H9c2 cardiomyocytes, and its inhibition protected these cells from Dox-induced senescence [30]. Activation of p38 MAPK leads to phosphorylation of the transcription factor cAMP-response element binding protein (CREB) on Ser-133, which promotes its binding to the promoter region of Redd1 to initiate its transcription [31]. Consistent with such mechanism, the present study showed that Dox-induced Redd1 upregulation in cardiomyocytes was blocked by inhibiting p38 MAPK. In turn, the fact that AAV9-mediated Redd1 overexpression abrogated the repressive effect of p38 MAPK inhibition on the Dox-induced senescence response reinforces the central role of Redd1 in cardiomyocyte senescence.

The signal transduction pathways that link senescence and inflammation in cardiac tissue are currently a topic of strong interest. NF-κB activation has been shown to participate in aging-associated cardiac pathologies [60–62]. Activation of NF-κB signaling, considered to be the main cause of chronic low-grade inflammation, is likely responsible for the increased manifestations of the SASP in the aging mouse heart [63, 64]. Lee et al. reported that Redd1 promoted atypical NF-κB activation by preventing the formation of the inactive NF-κB/IκBα complex through direct interaction with IκBα [22]. In line with previous studies, our study has shown that exogenous Redd1 overexpression promoted the phosphorylation levels of p65, along with the increase levels of p16^INK4a^ and p21, suggesting a major role of Redd1 in the regulation of NF-κB signaling and senescence-induced cardiac dysfunction. Our experiments showed that Redd1 silencing decreased p65 phosphorylation and nuclear translocation, which should lead to an anti-inflammatory effect. Conversely, we showed that exogenous Redd1 overexpression promoted the phosphorylation of p65. From these findings, we conclude that NF-κB inhibition might prevent or attenuate the senescence process triggered by Redd1 overexpression in cardiomyocytes. Thus, it would be highly interesting to determine the *in vivo* impact of NF-κB inhibition, alone or combined with Redd1 repression, in the cardiac aging process.

The present study has several limitations. First, most of the data hereby presented were obtained from *in vitro* and *in vivo* Dox-induced senescence models, which, as mentioned, may incompletely resemble the molecular events that mediate physiological aging. Second, although preliminary data was obtained regarding the influence of p38 and p65 signaling on the mechanisms by which Redd1 regulates cardiac senescence, further molecular analyses, as well as blocking experiments, are needed so that causal relationships could be fully established. In spite of these shortcomings, our findings suggest that therapeutic manipulation of cardiomyocyte Redd1 expression may be relevant to counteract cardiac dysfunction in old age.

## METHODS

### Recombinant viral vector construction

Adeno-associated virus 9 (AAV9) vectors carrying alternatively the mouse Redd1 gene (AAV9-Redd1), a shRNA targeting mouse Redd1 (AAV9-shRedd1), or the corresponding control sequences (i.e., AAV9-NC and AAV9-shNC), as well as lentivirus (Lv) carrying mouse Redd1 (Lv-Redd1), a shRNA targeting mouse Redd1 (Lv-shRedd1), or the corresponding control sequences (i.e., Lv-NC and Lv-shNC) were purchased from Weizhen Biotechnology Company (Shandong, China).

### Animal experiments

C57BL/6 male mice (6–8 weeks) were purchased from Beijing HFK Bioscience (Beijing, China). Mice were kept in the Tongji Medical School Experimental Animal Center and fed ad-libitum with chow diet and water. Animal experiments were carried out according to the Guide for the Care and Use of Laboratory Animals published by the NIH and approved by The Institutional Animal Care and Use Committee of Tongji Medical College, Huazhong University of Science and Technology. In the Dox-induced cardiomyocyte senescence model, Dox (D1515, Sigma-Aldrich, St. Louis, MO) was administered intraperitoneally via six injections (each containing 2.5 mg/kg Dox) over a period of 2 weeks, to reach a total cumulative dosage of 15 mg/kg [65, 66]. A separate group of mice administered an equivalent volume of normal saline served as controls. Viral vectors (AAV9-NC, AAV9-Redd1, AAV9-shNC, and AAV9-shRedd1) were delivered by tail vein injection 4 weeks before Dox administration. All mice were euthanized 4 months after the latest Dox/saline injection to evaluate the senescence phenotype. In the age-induced senescence model, C57BL/6 mice were divided into two groups: young (3~4 months old) and old (24 months old). Animal data were excluded from experiments based on pre-established criteria of visible abnormal tissue structure during sample harvest or other health issues, including any apparent wounds.

### Echocardiography

Four months after Dox administration, mice were anesthetized with 1.5% isoflurane and subjected to transthoracic echocardiography with a Vevo 2100 high-resolution micro imaging system (VisualSonics, Canada). Echocardiography images were acquired from long- and short-axis views. Left ventricular end-diastolic diameter (LVEDd) and left ventricular end-systolic diameter (LVESd) were measured in M-mode. Left ventricular fractional shortening (LVFS, %) and left ventricular ejection fraction (LVEF, %) were automatically calculated. All the parameters were acquired and averaged from six cardiac cycles.

### Histology and immunohistochemistry

Heart tissues were fixed with 4% paraformaldehyde, paraffin-embedded, and sectioned into 5 μm thick sections. Fibrosis was evaluated by Masson’s trichrome staining and calculated as the ratio of total collagen area to total area. Image-Pro Plus software (Media Cybernetics, MD, USA) was used to analyze the images. For IHC staining, slides were deparaffinized and subjected to heat-mediated antigen retrieval. After blocking in bovine serum albumin for 0.5 h, an anti-Redd1 antibody (1: 50 dilution; ab106356, Abcam, Cambridge, UK) was incubated for overnight at 4°C. A horseradish peroxidase-conjugated secondary antibody and 3, 3′-diaminobenzidine (DAB) were then used to detect immunoreactivity. Images (4–5 per LV section) were acquired at 200x magnification using a light microscope and analyzed using Image Pro Plus software.

### Cell culture and treatments

H9c2 rat cardiomyocytes were purchased from the American Type Culture Collection (ATCC, VA, USA) and cultured using Dulbecco's Modified Eagle's Medium (DMEM) supplemented with 10% fetal bovine serum, 1% penicillin, and 1% streptomycin at 37°C under an atmosphere of 5% CO_2_. Cells were treated at a density of approximately 80%, as reported previously [67]. H9c2 cardiomyocytes incubated with 0.1 μM Dox were harvested at different times (0, 24, 48, or 72 h), and senescence parameters assessed during the final 48 h of Dox incubation [30, 47, 68]. This time window was selected because explicit induction of senescence phenotypes in cardiomyocytes occur within this time frame. To examine the effect of increased or decreased expression of Redd1 on Dox-induced senescence, H9c2 cardiomyocytes were transduced with Lv-NC, Lv-Redd1, Lv-shNC, or Lv-shRedd1 and 48 h later treated with or without Dox (0.1 μM). SASP was evaluated 6 h after Dox administration, whereas p16^INK4a^ and p21 expression, as well as SA-β-gal activity, were studied 48 h after Dox administration. To study the involvement of p38 MAPK activation on Dox-induced upregulation of Redd1, H9c2 cardiomyocytes were pretreated for 30 min with or without 2 μM SB203580 (HY-10256, MCE, NJ, USA), a highly selective p38 MAPK inhibitor. Control experiments were performed by pre-treating cells with 0.1% DMSO, i.e., the vehicle used to dissolve both Dox and SB203580.

### Immunofluorescence

Immunofluorescent staining was performed as described elsewhere [69]. Briefly, deparaffinized slides were incubated with primary antibodies against p16^INK4a^ (1:50; ab54210, Abcam) and p21 (1:50; ab109199, Abcam) at 4°C overnight. After washing, fluorescein- and tetramethyl rhodamine-conjugated secondary antibodies (Jackson ImmunoResearch) were applied for 1 h at room temperature. Following DAPI staining, slides were mounted and three random fields per sample were photographed under epifluorescence microscopy. Relative fluorescence intensity was quantified with ImageJ software (NIH, MD, USA). To assess nuclear translocation of the NF-kB subunit p65, treated cells were fixed with 4% paraformaldehyde for 15 min, permeabilized with 0.5% Triton X-100 for 30 min, blocked in 10% goat serum for 15 min, and incubated with a rabbit monoclonal antibody against NF-kB p65 (1:50 dilution; 8242, CST, MA, USA) at 4°C in 1% goat serum overnight. After incubation with a fluorescein-labeled secondary antibody for 1 h, the cells were washed, stained with DAPI, and mounted. Immunofluorescence was recorded using epifluorescence microscopy at 400× magnification.

### Assessment of cellular senescence

Two different features of cellular senescence were investigated: i) senescence-associated β-galactosidase (SA-β-gal) activity, related to increased lysosomal biogenesis and function; and ii) expression of the cell cycle inhibitors p16^INK4a^ and p21 [29]. SA-β-gal staining was performed with a Senescence Cells Histochemical Staining kit (Sigma-Aldrich, MO, USA), according to the manufacturer’s protocol. Briefly, cultured H9c2 cardiomyocytes were fixed in 4% formaldehyde for 15 min at room temperature and then washed three times in phosphate-buffered saline (PBS) at room temperature. Slides were immersed in freshly prepared SA-β-gal staining solution and incubated overnight at 37°C without CO_2_. Stained sections were washed twice with PBS and senescence quantitated by visual inspection of blue/green stained cells with an inverted microscope (magnification, 200×). At least 6 fields of view were recorded for each experimental group. Expression levels of p16^INK4a^ and p21 were determined by both western blotting (see below) and immunofluorescence (already described).

### Western blotting

Total cellular proteins were extracted with RIPA lysis buffer applied at 4°C for 30 min and quantified using a BCA kit. Equal amounts of protein (60 μg) were separated by 10% SDS-PAGE, electro-transferred to a PVDF membrane, and blocked with 5% milk diluted in TBS/0.1% Tween-20 (TBST) buffer for 2 h at room temperature. Membranes were then incubated at 4°C overnight with the following primary antibodies: anti-β-actin (1:1000; 66009-1-Ig, Proteintech, Wuhan, China); anti-Redd1 (1:1000; ab10356, Abcam); anti-p16^INK4a^ (1:1000; ARG57377, Arigo, Taiwan, China); anti-p21 (1:1000; ab109199, Abcam); anti-NF-κB p65 (1:1000; 8242, CST, MA, USA); anti-phospho-NF-κB p65 (Ser468) (1:1000; 3039, CST); anti-p38 (1:1000; A10832, Abclonal, Wuhan, China); and anti-phospho-p38 (1:1000; AP0056, Abclonal). Following incubation with suitable HRP-conjugated secondary antibodies at room temperature for 2 h, the bands were visualized with a chemiluminescence detection system and quantified with Image J software.

### Quantitative real-time polymerase chain reaction (q-PCR)

Total cellular RNA was extracted with TRIzol reagent. Complementary DNA was synthesized using a PrimeScript RT Master Mix kit and then used for qPCR with SYBR Green Master Mixture on an ABI StepOnePlus RT-PCR system (Applied Biosystems, CA, USA). The cycling conditions were as follows: 95°C for 10 min, 40 cycles of 95°C for 15 s, and 60°C for 15 s. The qPCR was performed in 3 replicates of each sample. The β-actin housekeeping gene was used as a control. Relative gene expression levels were calculated by the 2^-ΔΔCq^ method [70]. The sequences of the primers used for q-PCR are listed in Table 1.

**Table 1:** Sequences of PCR primers.

**Genes**	**Forward**	**Reverse**
m-β-actin	5′- CGTTGACATCCGTAAAGACC-3′	5′- AACAGTCCGCCTAGAAGCA-3′
m-Redd1	5′- CAAGGCAAGAGCTGCCATAG-3′	5′- CCGGTACTTAGCGTCAGGG-3′
m-p16^INK4a^	5′- CGCAGGTTCTTGGTCACTGT-3′	5′- TGTTCACGAAAGCCAGAGCG-3′
m-p21	5′- CCTGGTGATGTCCGACCTG-3′	5′- CCATGAGCGCATCGCAATC-3′
m-IL-1β	5′-GCAACTGTTCCTGAACTCAACT-3′	5′-ATCTTTTGGGGTCCGTCAACT-3′
m-IL-6	5′-TAGTCCTTCCTACCCCAATTTCC-3′	5′-TTGGTCCTTAGCCACTCCTTC-3′
m-IL-12	5′-CTGTGCCTTGGTAGCATCTATG-3′	5′-GCAGAGTCTCGCCATTATGATTC-3′
m-TNF α	5′-CCCTCACACTCAGATCATCTTCT-3′	5′-GCTACGACGTGGGCTACAG-3′
r-β-actin	5′-GGAGATTACTGCCCTGGCTCCTA-3′	5′-GACTCATCGTACTCCTGCTTGCTG-3′
r-Redd1	5′-CACCGGCTTCAGAGTCATCA-3′	5′--CGGGTCTCCACCACAGAAAT-3′
r-p16^INK4a^	5′- GATGGGCAACGTCAAAGTGG-3′	5′- CCCAGCGGAGGAGAGTAGATA-3′
r-p21	5′- AGTCAAAGTTCCACCGTTCTCG-3′	5′- GCAAAGTATGCCGTCGTCTGT-3′
r-IL-1β	5′- GACAGAACATAAGCCAACAAG-3′	5′- GTCAACTATGTCCCGACCATT-3′
r-IL-6	5′- TGGAGTTCCGTTTCTACCTG-3′	5′- TTCATATTGCCAGTTCTTCG-3′
r-IL-12	5′-ACCCTCACCTGTGACAGTCC-3′	5′-TTCTTGTGGAGCAGCAGATG-3′
r-TNF α	5′- ACTCCCAGAAAAGCAAGCAA-3′	5′- CGAGCAGGAATGAGAAGAGG-3′

Abbreviations: Redd1: Regulated in development and DNA damage response-1; IL: interleukin; TNF α: tumor necrosis factor α.

### Statistical analysis

GraphPad Prism 8.0 (GraphPad Software Inc., CA, USA) was used for statistical analyses. Values are expressed as means ± SEM of at least three independent experiments. Unpaired, two-tailed Student’s *t*-test was used to assess differences between two groups. For multiple groups, significance was evaluated by one-way or two-way analysis of variance (ANOVA), followed by a post-hoc Tukey test. *P* < 0.05 was considered significant.

### Data availability

Data supporting the findings of this study are available from the corresponding author upon request.

## Supplementary Materials

Supplementary Figure 1
