# Supplementary material for: Redd1 knockdown prevents doxorubicin-induced cardiac senescence
Source: Aging (Albany NY). 2021 May 6;13(10):13788–806. doi: 10.18632/aging.202972 (PMC8202877; doi:10.18632/aging.202972)
Supplement: Supplementary Figure 1 [file aging-13-202972-s001.pdf]

SUPPLEMENTARY FIGURE

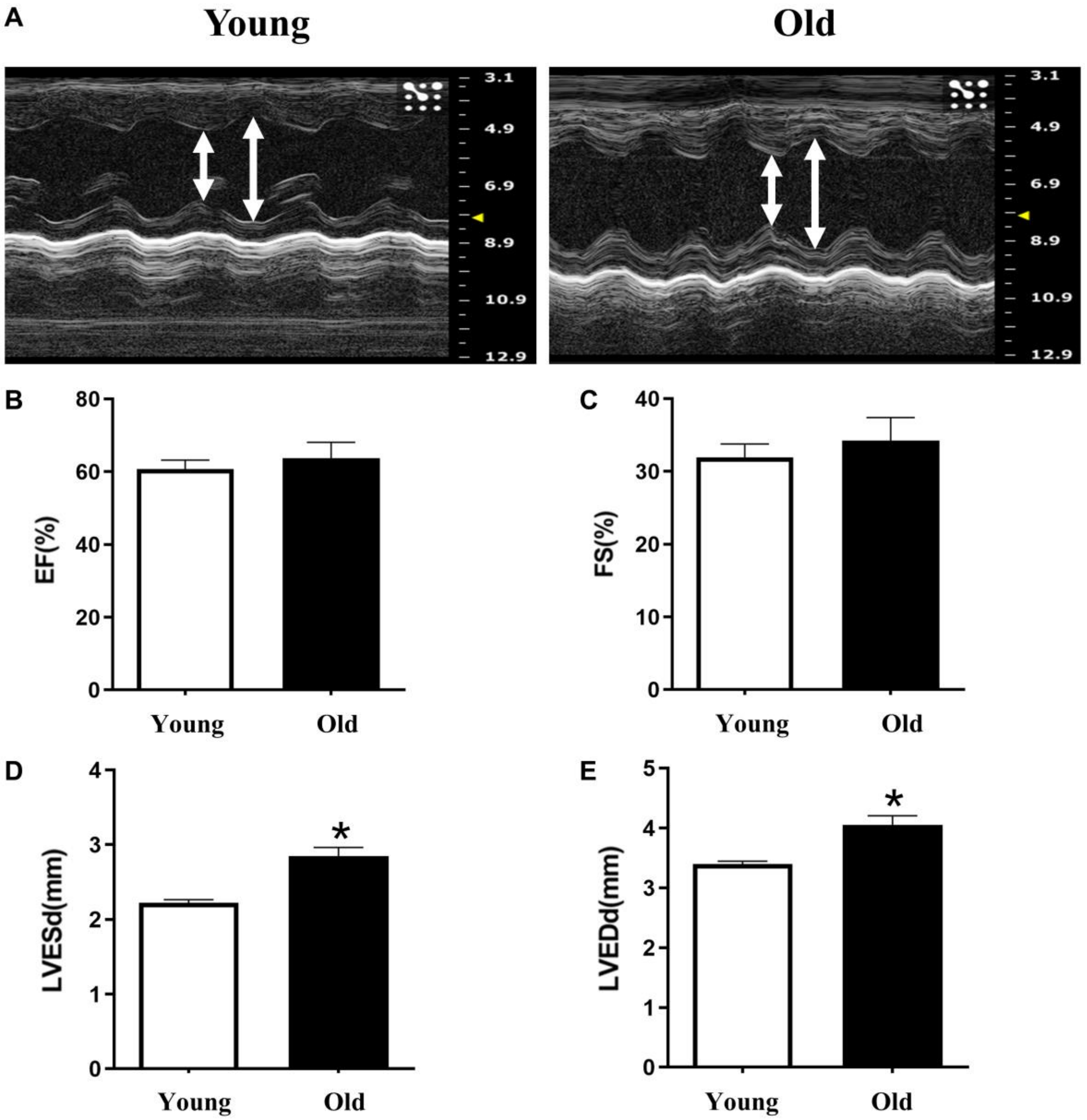

Supplementary Figure 1. Aging induces cardiac dysfunction in mice. (**A**) Representative echocardiography photographs depicting heart function in the different experimental groups. (**B**–**E**) Quantitative analysis of heart function, as determined by percent fractional shortening (FS%), ejection fraction (EF%), left ventricular (LV) end-systolic diameter (LVESd), and LV end-diastolic diameter (LVEDd) (n = 6 mice per group). Data are mean ± SEM. $^{*}p < 0.05$ vs. young group.
